# The PEDIAVALVE project – tissue-engineered pulmonary valves for paediatric valvular replacement

**DOI:** 10.3389/fbioe.2026.1902783

**Published:** 2026-09-10

**Authors:** Ionela Cotfas, Hussam Al Hussein, Hamida Al Hussein, Klara Brânzaniuc, Ovidiu Simion Cotoi, Dan Simionescu, Marius Harpa

**Affiliations:** 1 Regenerative Medicine Laboratory, George Emil Palade University of Medicine, Pharmacy, Science, and Technology of Targu Mures, Targu Mures, Romania; 2 Doctoral School of Medicine and Pharmacy, I.O.S.U.D., George Emil Palade University of Medicine, Pharmacy, Science, and Technology of Targu Mures, Targu Mures, Romania; 3 Department of Bioengineering, Clemson University, Clemson, SC, United States

**Keywords:** decellularization, extracellular matrix, heart valve, paediatric heart valve, regenerative medicine, tissue engineering

## Abstract

Current paediatric options for pulmonary heart valve (PHV) substitutes are imperfect, requiring numerous reinterventions until maturity. Substitutes with a diameter under 15 mm have a limited service life, underscoring the critical need for small-diameter valves. To address this need, we obtained and tested decellularized, DNA-depleted, small-diameter valvular substitutes using tissue-engineered lamb PHVs. Lamb PHVs were subjected to a decellularization perfusion protocol, yielding a 3D extracellular matrix (ECM)- derived acellular scaffold. Sterility, residual nuclei (histology) and DNA content were assessed. Hemodynamic performance was assessed in a dedicated heart valve bioreactor, compared with freshly harvested PHV. Cytocompatibility was assessed using fibroblasts and endothelial cells, and the *ex vivo* surgical implantation feasibility was evaluated. Decellularization validation revealed preserved ECM without cell nuclei, and over 90% DNA reduction. *In vitro* hemodynamic assessment showed physiological behaviour with complete opening and no evidence of regurgitation or stenosis; geometric and Doppler parameters were comparable to fresh valves. Cells remained viable at 3, 7 and 14 days, and *ex vivo* implantations were successful, with adequate suturing. Decellularization of small ovine PHV is feasible, yielding cytocompatible scaffolds with good hemodynamics and surgical handling. Although initial results are promising, further quantitative characterization and *in vivo* validation are needed before clinical translation.

## 1 Introduction

### 1.1 The right ventricular outflow tract (RVOT)

The RVOT is the right ventricular anatomical component situated between the supraventricular crest and the pulmonary trunk root. It is a muscular tubular segment that carries blood from the right ventricle into the pulmonary circulation. Its particular architecture–the septal component forming its posterior-medial wall and the funnel-shaped sub-pulmonary infundibulum and their thickness variances represent the causes of their congenital remodelling (Jakiel et al., 2025).

### 1.2 The RVOT is often affected by congenital diseases

Literature describes a series of various congenital conditions that involve the RVOT, at different levels such as pulmonary stenosis, double-chambered right ventricle, pulmonary atresia, truncus arteriosus, malposition of the large arteries and tetralogy of Fallot (Heaton et al., 2024; Loukas et al., 2013; Horenstein et al., 2024).

### 1.3 Treatment options for RVOT-affected congenital diseases

The main surgical approach in these patients’ groups is the RVOT reconstruction with pulmonary valve replacement. Following the surgical intervention, the patient may remain with a patched RVOT and chronic pulmonary regurgitation or undergo the implantation of a right ventricular-to-pulmonary artery conduit (Contegra conduit) (Geva et al., 2024; Morray et al., 2017). The time-dependent tissue degeneration and the somatic growth of the surgical reconstruction regularly progress to stenosis, regurgitation or both, leading to multiple pulmonary valve replacements over the patient’s lifetime (Geva et al., 2024).

### 1.4 Limitations of current options

The central drawback is that none of the options has lifelong durability, and none can grow with the patient, with reinterventions expected and part of the management plan. Recent literature reviews expose the pulmonary homografts as a reliable option, and the bovine jugular vein conduits (Contegra) reveal comparable durability in selected groups, having the advantage of a broad size availability (Qian et al., 2021; Brown et al., 2006). Decellularized pulmonary homografts were developed to reduce immunogenicity and grow with the patient. The ESPOIR trial illustrated greater freedom from explantation and decreased valve degeneration at a 10-year interval compared to cryopreserved homografts or bovine jugular vein conduits (Boethig et al., 2019).

### 1.5 Patients aged less than 2 years are the most difficult environment

Patients in need of RVOT reconstruction represent a large proportion, between 3 and 4 months old and teens. This aspect is important due to the large variation of needed valvular implants. 12- to 14-mm valves are required for younger patients, whereas older patients require larger implants. The literature indicates that conduits above 15 mm perform better than those below 14 mm (Fiore et al., 2011; Gist et al., 2012). A particular challenge in these paediatric groups is represented by the children aged under 2 years old. They experience rapid somatic development, leading to early prosthesis outgrowth. The new generation of regenerative valvular substitutes should be able to grow with the recipient and remain viable.

## 2 Materials and methods

### 2.1 Lamb hearts

This article is part of a research grant that has the Ethics Committee approval from George Emil Palade University of Medicine, Pharmacy, Science, and Technology of Târgu Mureş. Lamb hearts (male and female, aged 6–8 months, weighing 10–15 kg) were obtained from a local abattoir and rapidly transported in ice water into the University’s Regenerative Medicine Laboratory. Using a basic surgical instrument set, the pulmonary artery trunk was harvested, maintaining about 3–4 cm subvalvular myocardium and the tubular part of the pulmonary artery, below its division in the pulmonary arteries, method previously described (Harpa et al., 2015).

### 2.2 Decellularization of lamb pulmonary arteries

**FIGURE 1:**
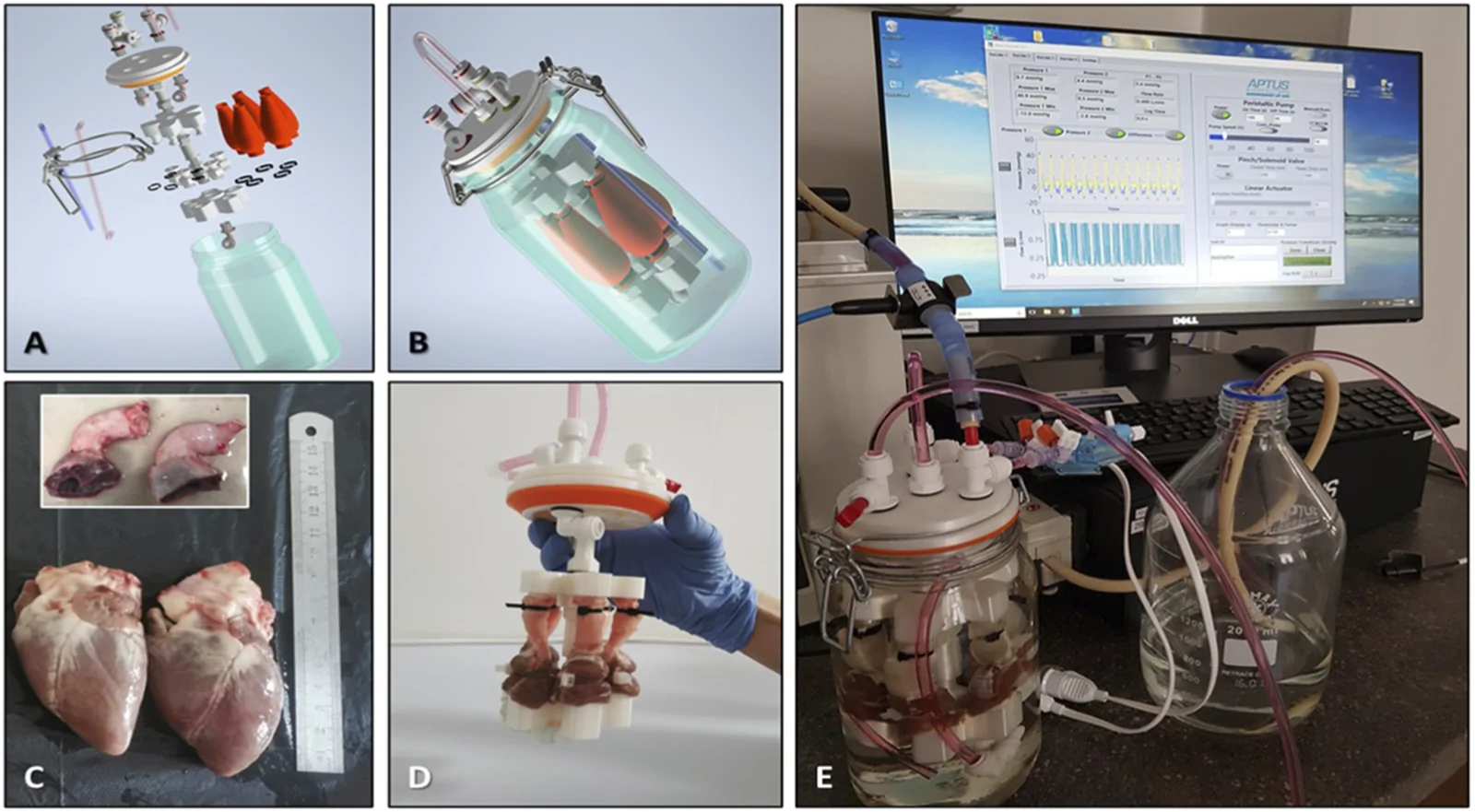
Design and implementation of the decellularization system. **(A,B)** The system was first designed in AutoCAD (Autodesk, CA, USA), and then all components were printed on a 3D printer. The system contains mounts for 5 valves and is fixed on a glass container. **(C)** Lamb hearts were brought to the laboratory. **(D)** The valves were dissected and mounted in the system, after which the container was connected to a perfusion pump with pressure and fluid flow measurement via a LabView (NI, TX, USA) interface **(E)**.

The isolated pulmonary arteries were then subjected to a perfusion-based decellularization protocol to obtain valvular acellular biological scaffolds. Lambs' valves were placed in a manufactured perfusion decellularization system (Figure 1) composed of a plastic tubular support with 5 branches, one for each valve. Valves were mounted on the lid of the box using zip ties, ensuring maximal visibility while facilitating valve mounting and handling. The decellularization solution infusion was performed through a tubing system attached to a peristaltic pump with a 3 min on/30 s off on a cycle regimen. Each valve was decellularized separately under an inner-outer pressure gradient. Pressures and flow were constantly monitored with pressure transducers and a digital flowmeter. Monitoring and control were achieved by the computer through the LabVIEW interface.

The decellularization protocol consisted of a 7-day-long treatment with physical, chemical and enzymatic agents, a more concise and adapted version of the previous published protocols (Sierad et al., 2015; Movileanu et al., 2019; Movileanu et al., 2021). The protocol started with 0.02% Sodium Azide (Sigma-Aldrich Chemistry, St. Louis, USA) for the first 24h. Next, the valves were exposed to 0.05 M NaOH (Lach-Ner, Neratovice, Czech Republic) for 2h and after a water rinse, the Decell solution (SDS - sodium dodecyl sulfate (Sigma-Aldrich Chemistry, St. Louis, USA), DOC deoxycholate (Sigma-Aldrich Chemistry, St. Louis, USA), Triton X100(PanReac AppliChem, Spain)) was inserted and changed every 48 h for the next 6 days of the protocol. The decell solution was rinsed with water, and the Sodium azide was introduced overnight. For the next 6 days, the lamb pulmonary valves were exposed to a solution of DNA-ase/RN-ase (PanReac AppliChem, Spain) at 37 degrees Celsius, changed every 48h. Short treatment with 70% Ethanol followed and the sterilization with Peracetic Acid (Merck KGaA, Darmstadt, Germany) was the final step. All the previous exposed steps were performed under a continuous pulsatile flow under a pressure gradient of 4–5 mmHg.

Decellularization validation was performed by two complementary methods: histological analysis using (1) cryosections with DAPI (4′,6-diamidino-2-phenylindole) nuclear staining, respectively Hematoxylin-Eosin stain and (2) DNA extraction and analysis with detection of DNA presence on Ethidium Bromide Agarose gel electrophoresis. For these tests, tissue samples were collected from three essential valvular areas: the leaflets, the valvular sinus and the arterial wall. Additionally, sterility of the valves at the protocol finale was aimed for; for this purpose, a sample was harvested from each valve and microbiologically tested, as previously described (Movileanu et al., 2021).

For histology, samples from the three areas were harvested, and cryosections were stained with DAPI (Thermofisher, Massachusetts, USA) and examined under fluorescent light. For nucleic acid analysis, approximately 25 mg of tissue samples were weighed and DNA was extracted using the Qiagen kit (Qiagen N.V., Venlo, the Netherlands). Purified DNA was quantified on NanoDrop (Thermo Fisher Scientific, MA, USA) and applied to an Ethidium Bromide Agarose gel for migration and analysis. Quantitative results were expressed as ng DNA/mg tissue. All these analyses were also performed on freshly harvested, native, unexposed valve samples.

Compositional analyses of individual extracellular matrix constituents (collagen, elastin, glycosaminoglycans, laminin, and fibronectin) were not performed in the present study; matrix assessment focused on preservation of the three-dimensional architecture and functional geometry.

### 2.3 Functional tests of acellular ovine RVOT

Tissue-engineered heart valves were *in vitro* functionally tested by using a dedicated heart valve bioreactor (Aptus Bioreactors, SC, USA). This assembly is a manufactured machine that exposes the valves to regimens that replicate the heart’s hemodynamics in the laboratory (pressure gradients, heart rate, stroke volume and cardiac output). Using the bioreactor, the tissue-engineered valves (n = 8) were compared to freshly harvested lamb pulmonary valves (n = 8) used as controls. The two-group comparison consisted of two sets of analyses–geometric (image-based) and echocardiographic–performed in a controlled environment, ensuring similar testing conditions for both valve groups.

Pulmonary circulation conditions (systolic and diastolic pressures, cardiac output, heart rate) were replicated in the lab using a specialized heart valve bioreactor. The studied valves were sequentially mounted in the bioreactor, and for each valve, a series of 12 opening/closing cycles was captured using a high-frame-rate camera. Using a clinical ultrasound machine equipped with a cardiac ultrasound transducer commonly used for transthoracic evaluation (3.5 MHz), echocardiographic assessment was performed.

The statistical comparison between the two groups was made between the parameters analyzed during the Continuous Doppler examination–Maximal Velocity (Vmax), Medium Velocity (Vmed), Maximal Pressure (Pmax), Mean Pressure (Pmed), Velocity Time Integral (VTI). The averages of the 5 parameters, calculated for each valve individually, were compared across the 12 cycles.

### 2.4 The heart valve bioreactor

Using the Aptus heart valve bioreactor, the two valve groups were cyclically exposed to hemodynamic conditions of the pulmonary circulation in our laboratory. This system was created and implemented internally. It has an acrylic structure and three main compartments: an aortic chamber (up), a ventricular chamber (middle), and a basal air chamber, separated from the middle one by a rubber membrane.

The membrane, powered by an air pump, displaces fluid from the middle (ventricular) chamber into the upper (aortic) chamber through the valve. When the air pump lowers the air chamber pressure at the cycle end, the membrane descends, the ventricular chamber pressure drops, and the valve closes as a result. Multiple access ports are included in each of the three compartments for pressure measurements and monitoring, sterile handling during culture media exchange and oxygen source connections. The bioreactor has a working capacity of 750 mL of liquid media. The aortic chamber has specifically designed walls to enable photo and video recording, at the same time permitting ultrasound passage proper for echography examinations.

Operating parameters and internal environment characteristics were continuously monitored and analyzed in order to provide a similar testing environment. During the tests carried out within the project, the hemodynamic conditions of the pulmonary circulation were recreated: systolic/diastolic pressure 20/5 mmHg, closing-opening frequency 60 bpm, cardiac output 5 L/min.

### 2.5 Geometric assessment

**FIGURE 2:**
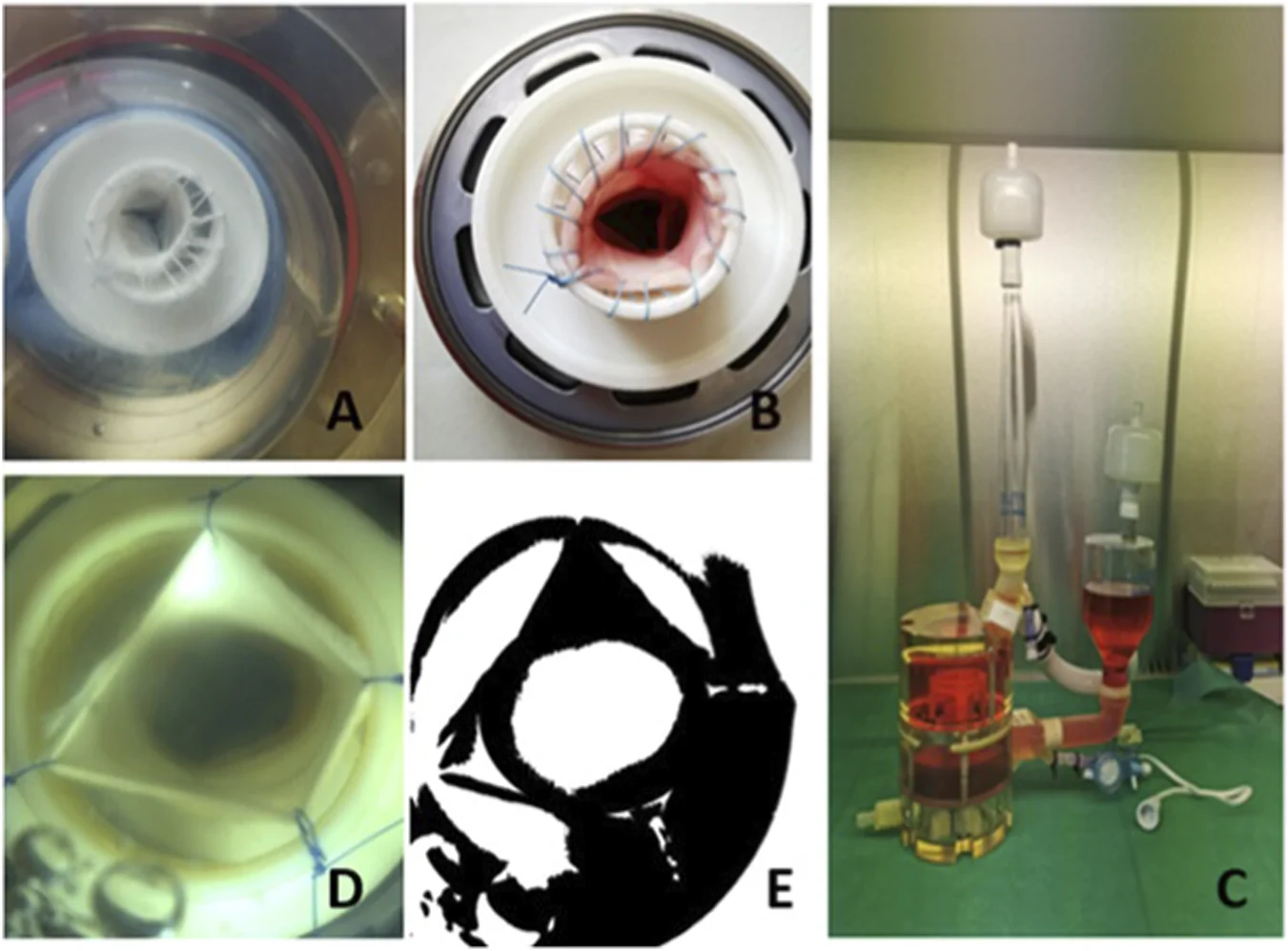
Mounting the valves in the bioreactor–**(A)** Decell valve, **(B)** fresh valve, **(C)** heart valve bioreactor, **(D)** image captured by the high-speed camera, **(E)** image processed with the ImageJ Software.

Successively, the 16 valves were placed in the bioreactor using 2.0 Ticron sutures for anchoring in the stabilization support (Figure 2). The camera was placed at the level of the bioreactor’s upper wall and mounted on a support manufactured with a 3D printer. Video recordings of 12 consecutive closing-opening cycles were made for each valve. Each cycle was analysed individually, extracting individual photo frames (an average of 18 frames/cycle - maximum 23 f/c, minimum 14 f/c). Each image thus obtained was processed in ImageJ, calculating the area of the valve orifice on all snapshots captured up to the maximum value. In total, 3140 images showing the opening and closing of the valves were examined. An example is presented in Table 1. Using a high-speed camera, multiple sequences were analyzed, measuring the maximal opening area.

**TABLE 1:** Exemplification of the number of frames extracted from a recorded video for a decell valve and the measured opening area.

1st cyc	​	2nd cyc	​	3rd cyc	​	4th cyc	​	5th cyc	​	6th cyc	​
Frame	A mm2	Frame	A mm2	Frame	A mm2	Frame	A mm2	Frame	A mm2	Frame	A mm2
10	4.464	87	1.954	163	1.765	239	3.73	315	7.079	390	1.844
11	25.768	88	37.211	164	13.611	240	23.017	316	34.688	391	14.749
12	46.8	89	50.322	165	40.824	241	47.072	317	46.924	392	42.269
13	43.159	90	46.934	166	48.722	242	49.651	318	48.854	393	42.61
14	39.783	91	39.057	167	39.668	243	40.142	319	47.574	394	39.831
15	38.638	92	34.345	168	35.909	244	38.958	320	42.911	395	39.907
16	33.65	93	32.034	169	34.751	245	32.422	321	38.687	396	34.581
17	32.224	94	31.87	170	35.601	246	31.318	322	33.293	397	30.131
18	32.332	95	33.203	171	31.73	247	31.738	323	33.433	398	32.187
19	31.378	96	29.278	172	30.837	248	28.986	324	27.806	399	27.43
20	26.952	97	25.067	173	28.045	249	25.288	325	22.411	400	22.179
21	23.076	98	22.274	174	22.985	250	20.24	326	20.421	401	20.225
22	17.493	99	17.28	175	19.662	251	23.037	327	19.584	402	17.954
23	17.747	100	16.935	176	19.897	252	21.376	328	18.67	403	17.194
24	8.749	101	7.402	177	14.8	253	17.352	329	13.394	404	8.139
25	3.206	102	3.369	178	7.295	254	13.909	330	5.007	405	4.731
​	​	​	​	179	3.138	255	1.666	331	2.481	406	2.831

### 2.6 Echocardiography assessment

Using a clinical ultrasound machine (Mindray, Shenzhen, China) in clinical practice, the behavior of the valves in the bioreactor was analyzed, with the bioreactor walls made of material that allowed ultrasound to pass. A two-dimensional morphological characterization of the valves was performed (the appearance of the components of the valve apparatus, their integrity, mobility, degree of opening, maturation), and the valve functionality was evaluated by the degree of competence using Color Doppler and the analysis of trans-valvular velocities with the determination of gradients, upon Continuous Doppler interrogation in order to identify any existing valve stenoses.

Additionally, echocardiographic assessment was performed for 3D-printed mechanical valve and biological valve prosthesis, serving as controls, to evaluate their “in the bioreactor” behavior and echocardiographic functionality.

The two groups of valves studied were placed in the bioreactor in succession, with 12 closing-opening cycles recorded. For each closing-opening cycle, using the Continuous Doppler curve, the Maximum Velocity (Vmax), Average Velocity (Vmed), Maximum Pressure (Pmax), Average Pressure (P Med) and the Velocity-Time Integral (VTI) were calculated.

### 2.7 Biostatistical analyses

Statistical analyses were conducted using GraphPad (GraphPad Software, CA, USA). Comparisons between the two groups were performed using an unpaired two-tailed Student’s t–test. A p-value under 0.05 was considered indicative of statistical significance. Statistical analyses were used to compare the maximal opening geometric area and the valve echography functional performances.

### 2.8 Cytocompatibility testing of acellular ovine RVOT

To carry out the cytocompatibility testing, human endothelial cells and human fibroblasts were grown in culture (37 °C, 5% CO_2_) and seeded (200,000 per cm2) on pieces of tissue isolated from the cusp, wall and sinus of decellularized pulmonary valves. The samples were placed in wells of 6-well plates and cultured for up to 14 days, with medium changes every 3 days. Tissue samples were harvested at 3, 7 and 14 days and analyzed by Live/Dead staining and fluorescence microscopy. Cytocompatibility was assessed as an initial viability screen, following the principles of ISO 10993–5, over an extended 14-day period; quantitative proliferation, infiltration, and phenotypic analyses were not performed at this stage.

### 2.9 Mock implantation of tissue-engineered pulmonary valves

For this purpose, the cardiovascular surgical collaborators from the Heart Institute of Târgu Mureş were asked to handle the decellularized ovine valves and implant them *ex vivo* in sheep hearts using standard surgical technique. The following were tested: general appearance, ease of handling, size matching, and the feasibility of suturing to the pulmonary valve annulus.

## 3 Results

### 3.1 Pulmonary valves decellularization

**FIGURE 3:**
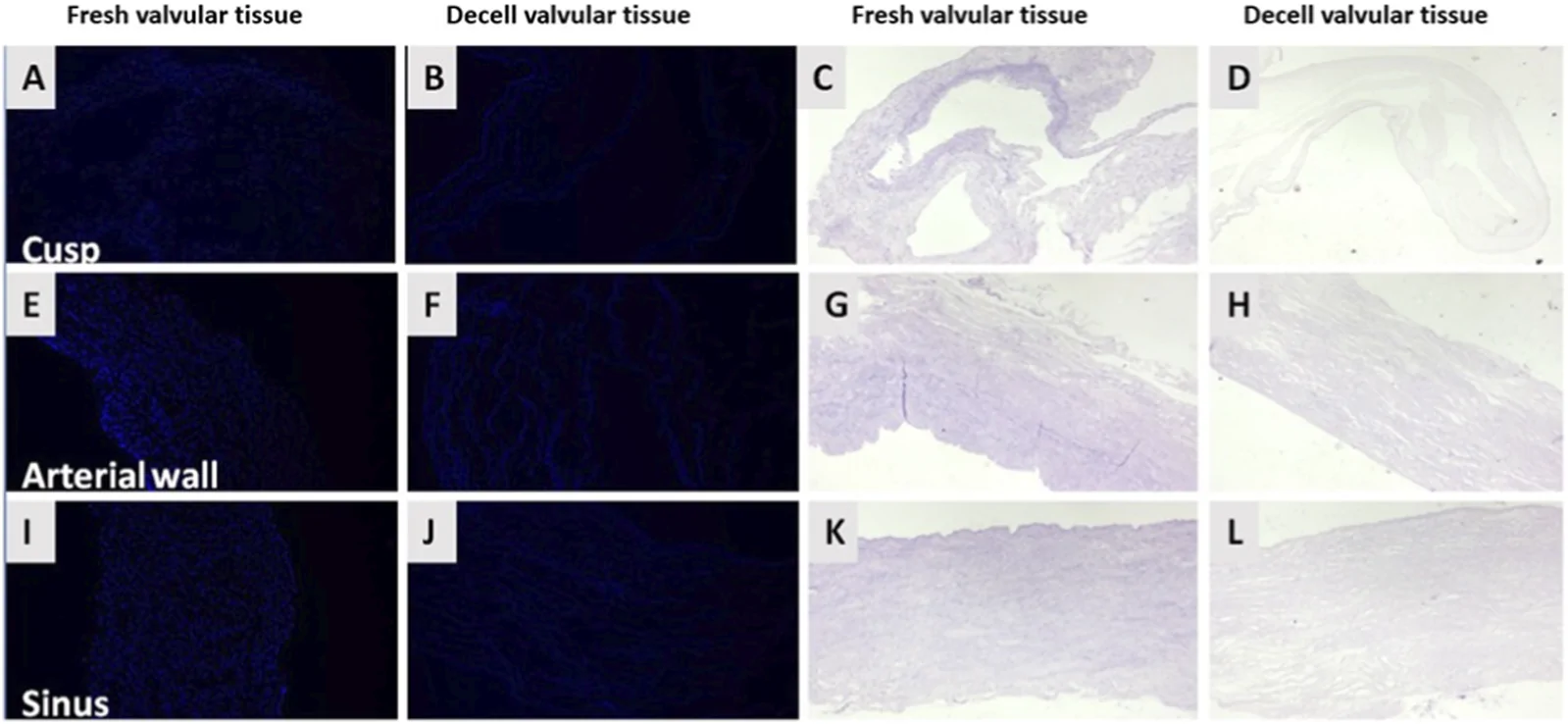
Validation of decellularization of ovine pulmonary valves. Samples from cusps, wall and sinus. DAPI staining **(A,B,E,F,I,J)**, H&E staining **(C,D,G,H,K,L)**, cell nuclei present in fresh tissue (left columns), nuclei absent after decellularization (right columns).

25 ovine lamb pulmonary valves underwent decellularization, resulting in 25 decellularized heart valves. Qualitative assessment of the decellularization exposed sterile decellularized valves and DAPI and hematoxylin-Eosine staining of the valvular structures (arterial wall, leaflet, valvular sinus) validated the absence of cellular nuclei (Figure 3).

**FIGURE 4:**
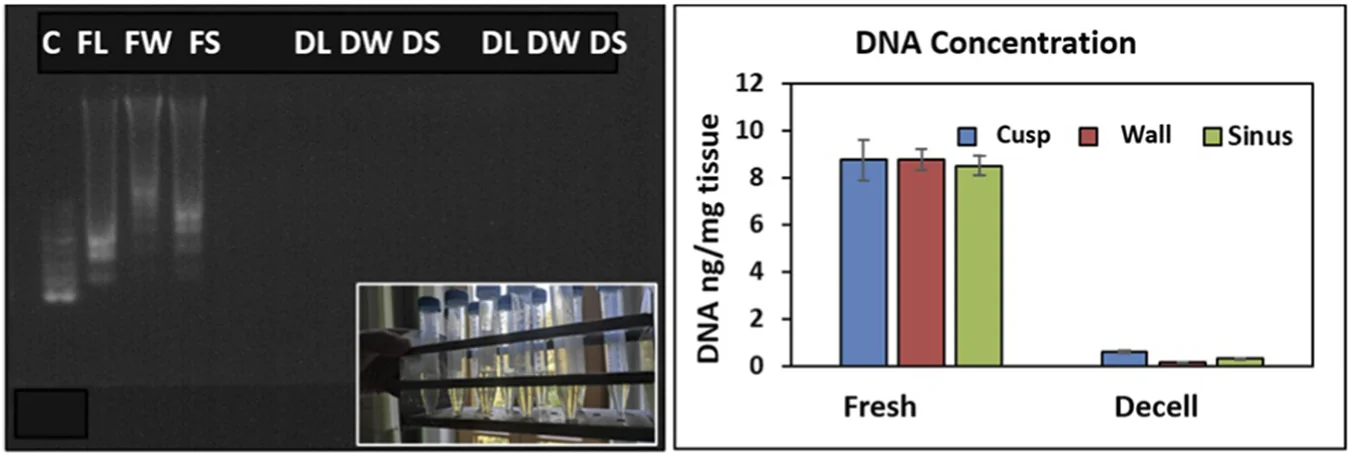
The same tissue samples were used for DNA extraction, and the extracts were analyzed on agarose (left) and by spectrophotometry (right). Both tests show a decrease in the amount of DNA below the detection limit after decellularization. Inset in left: 100% sterility for all samples. Legend for the left image: C = DNA standard, F = fresh pulmonary valves, D = decellularized pulmonary valves, L = leaflet, W = wall, S = sinus.

Quantitative measurement confirmed the microscopy results, revealing the absence of detectable DNA bands on agarose gel electrophoresis, respectively, a 93% reduction of leaflet DNA, 98% of the arterial wall and 96% in the valvular sinus (Figure 4).

In our previous studies, the same results were validated but on larger ovine pulmonary valves harvested from adult animals (Movileanu et al., 2019; Movileanu et al., 2021). This shortened and adapted decellularization protocol proved efficient at removing cells while preserving the 3D extracellular matrix.

### 3.2 Hemodynamic behaviour

#### 3.2.1 Geometric analysis

**FIGURE 5:**
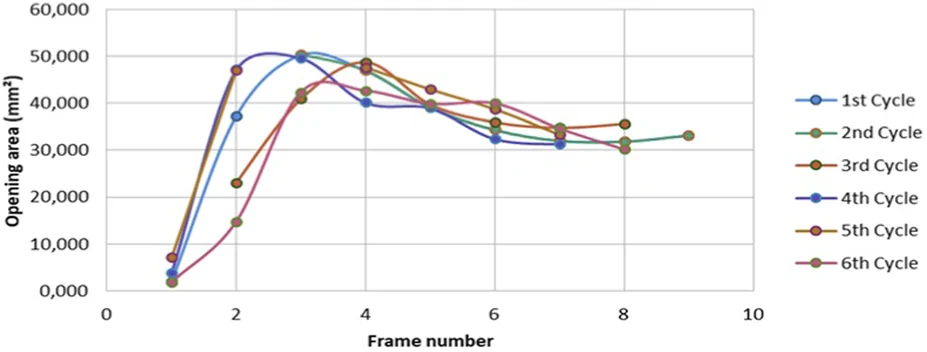
Frame-by-frame opening area (mm^2^) of a decellularized pulmonary valve over six consecutive cycles in the bioreactor. Orifice area was measured on individual frames extracted from high-speed camera recordings and processed in ImageJ. Peak opening values ranged between 47 and 51 mm^2^, with consistent behaviour across all cycles.

Over 3140 images at the opening and closing points were examined, as shown in Figure 5; Table 1, which illustrate the values obtained from 1 valve.

The statistical comparison of decellularized lamb pulmonary valves and fresh controls in terms of average maximal opening valvular area revealed a p-value of 0.193, statistically insignificant (Table 2). The decellularized valves showed no significant difference in maximal valvular area compared to the controls.

**TABLE 2:** Descriptive statistics of the comparison of geometric opening area in the decell and fresh valve groups.

*Decell valves*	​	*Fresh valves*	​
Mean	50.18025	Mean	51.7335
Standard error	6.905493643	Standard error	4.137224078
Median	46.888	Median	49.629
Mode	#N/A	Mode	#N/A
Standard deviation	19.53168553	Standard deviation	11.7018368
Sample variance	381.4867396	Sample variance	136.9329846
Kurtosis	1.698621972	Kurtosis	−1.137073305
Skewness	0.506611652	Skewness	0.138655749
Range	67.901	Range	33.135
Minimum	18.637	Minimum	34.59
Maximum	86.538	Maximum	67.725
Count	8	Count	8
CV	39%	​	23%
P Value	0.1930	​	​

#### 3.2.2 Echocardiography evaluation

Approximately 200 such recordings were analyzed.

From a qualitative point of view, the valves in both study groups showed physiological movements of the valve components (cusps, sinuses, walls), with complete opening of the cusps, without signs of restriction and without highlighting the presence of continuity solutions at the level of the valve apparatus. Color Doppler ultrasound revealed no regurgitation jets, highlighting preserved valve competence.

**FIGURE 6:**
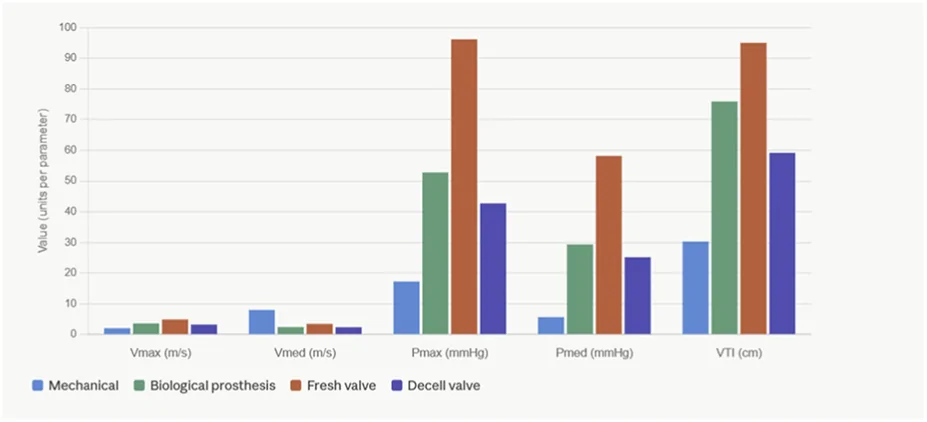
Mean Doppler parameters measured over 12 cycles for the four valve types evaluated in the bioreactor. The decellularized valve performed similarly to the biological prosthesis across all parameters, and differences relative to the fresh valve did not reach statistical significance for Vmax (p = 0.053), Pmax (p = 0.158), or VTI. Vmax—maximal velocity (m/s); Vmed—mean velocity (m/s); Pmax—maximal pressure gradient (mmHg); Pmed—mean pressure gradient (mmHg); VTI—velocity-time integral (cm).

Focused on the values ​​determined with Continuous Doppler, the group of decellularized valves presented statistically significantly lower Vmed (p = 0.035) and statistically insignificantly Vmax, Pmax, Pmed and VTI, when averages compared (Figure 6; Tables 3, 4).

**TABLE 3:** *In vitro* (in the heart valve bioreactor), echography analysed the behaviour of standard heart valve substitutes, a fresh lamb pulmonary valve, and a decellularized lamb pulmonary heart valve. The used Continuous Doppler parameters, as follows: Maximal Velocity (Vmax), Medium Velocity (Vmed), Maximal Pressure (Pmax), Mean Pressure (Pmed), Velocity Time Integral (VTI).

Mechanical valve	Cycle #1	#2	Cycle #3	Cycle #4	Cycle #5	Cycle #6	Cycle #7	Cycle #8	Cycle #9	Cycle #10	Cycle #11	Cycle #12
Vmax	1.985	2.084	2.169	2.224	1.871	1.871	1.97	2.311	2.016	2.174	2.124	2.045
Vmed	10.446	12.373	12.054	8.064	6.703	7.109	6.666	9.189	6.642	6.46	6.583	3.76
Pmax	15.76	17.36	18.82	20.07	14.01	14.01	15.54	21.36	16.26	18.91	18.06	16.74
Pmed	5.93	7.09	7.27	6.69	5.04	5.16	5.58	6.54	5.14	5.05	4.88	3.64
VTI	38.86	42.31	45.56	35.32	24.93	26.44	23.6	30.88	23.31	24.03	27.46	21.43
Biological prosthesis												
Vmax	3.299	3.261	3.203	3.337	3.357	4.009	3.951	3.932	3.913	3.875	3.625	3.683
Vmed	2.455	2.497	2.408	2.011	2.075	2.463	2.713	2.54	2.472	2.467	2.517	2.483
Pmax	43.55	42.57	41.05	44.57	45.08	64.3	62.47	61.86	61.26	60.06	52.58	54.26
Pmed	28.9	28.82	26.32	22.19	23.59	31.8	35.16	32.64	31.1	31.01	30.67	30.24
VTI	72.19	67.42	60.69	71.2	68.5	85.72	84.67	83.83	81.59	78.47	78.54	77.47
Fresh valve												
Vmax	4.891	5.283	5.087	4.957	4.731	4.989	4.859	4.793	4.598	4.663	5.054	4.859
Vmed	3.587	3.874	3.311	3.393	3.412	3.318	3.498	3.378	3.012	3.615	3.934	3.363
Pmax	95.72	111.65	103.53	98.29	90.68	99.59	94.45	91.93	84.58	86.99	102.21	94.45
Pmed	60.89	68.98	56.33	55.57	56.31	54.39	58.69	55.22	45.61	59.04	71.89	55.07
VTI	92.82	104.61	97.36	93.67	90.11	101.55	94.46	95.26	93.98	88.95	96.78	90.81
Decell valve												
Vmax	3.537	3.331	3.261	3.411	3.135	3.136	3.16	3.311	3.361	3.085	2.985	3.185
Vmed	2.764	2.397	2.402	2.408	2.058	2.363	2.301	2.531	2.353	2.277	2.231	2.35
Pmax	50.04	43.86	42.54	46.56	39.33	47.23	39.96	43.86	45.2	38.08	35.65	40.6
Pmed	32.43	25.81	25.2	25.8	21.32	25.77	23.73	28.04	25.33	23.06	22.23	24.07
VTI	59.7	58.97	60.55	62.14	56.8	60.97	58	60.76	63.55	58.75	50.87	57.81

**TABLE 4:** Results of the statistical analysis of average Vmax, Vmed, Pmax, Pmed, VTI of decellularized and fresh lamb pulmonary valves.

Averaged parameter	Statistical comparison (p)
Vmax	0.053
Vmed	0.035
Pmax	0.158
Pmed	0.079
VTI	0.13

### 3.3 Cytocompatibility

**FIGURE 7:**
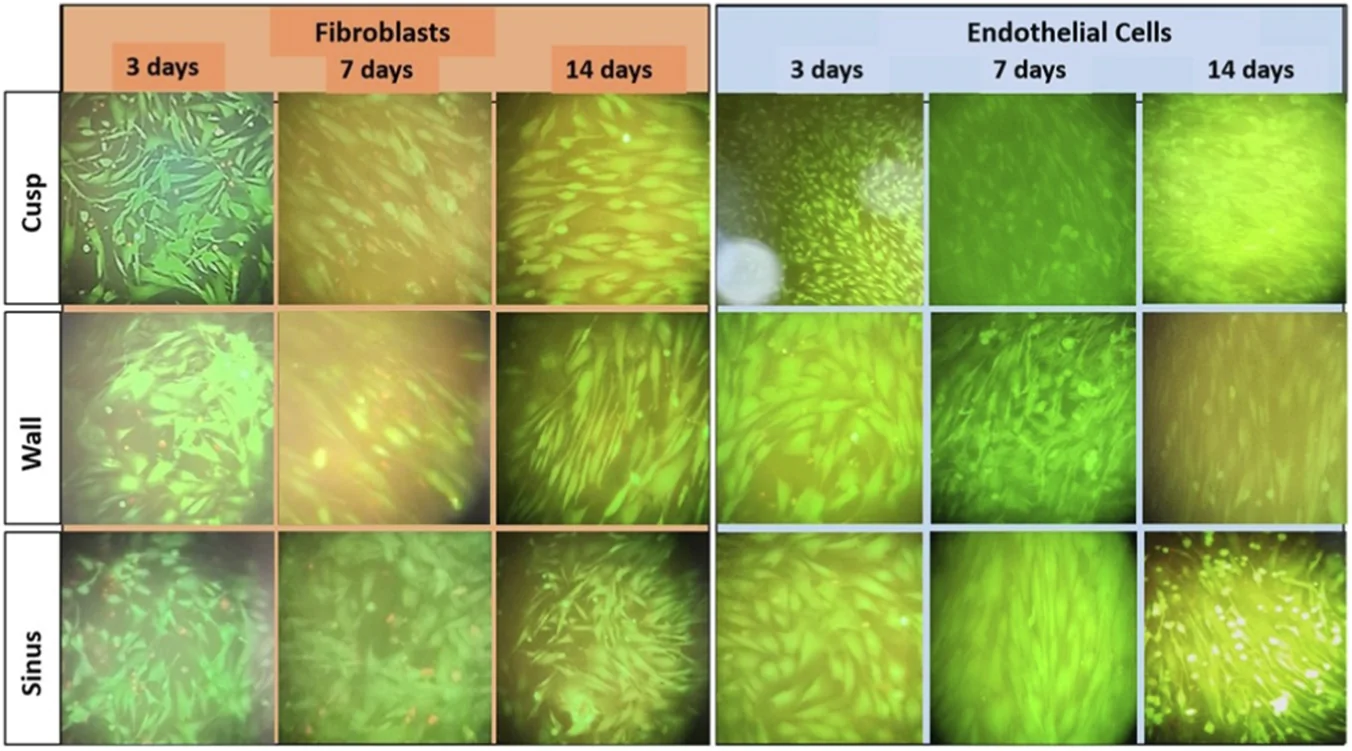
Biocompatibility testing of decellularized pulmonary valves, Live/Dead staining after seeding with fibroblasts and endothelial cells. Cells appeared viable 14 days after seeding.

All valvular surfaces demonstrated cytocompatibility, with endothelial cells and fibroblasts exhibiting normal adhesion and proliferation characteristics after 14 days (Figure 7). Considering that ISO 10993–5 only requires 72 h of incubation, this study demonstrates cytocompatibility over an extended period.

### 3.4 Mock implantation

**FIGURE 8:**
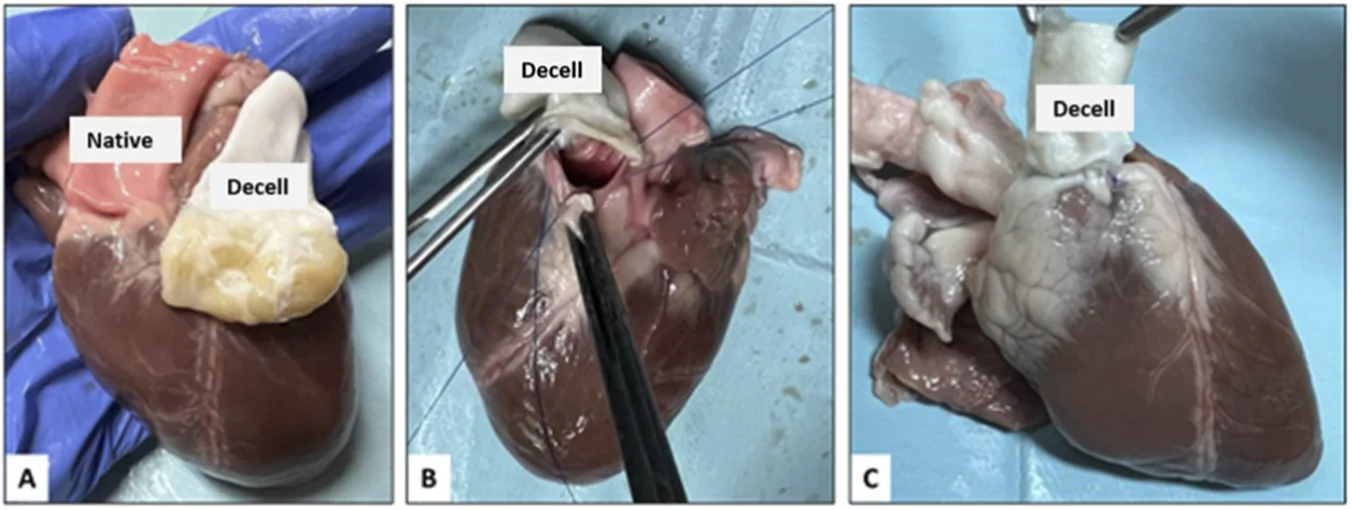
*Ex vivo* implantation of the decellularized pulmonary valve (decell). **(A)** A sheep heart with right ventricular outflow tract (RVOT) before “surgery”; a decellularized valve was placed next to the RVOT for comparison. **(B)** The native valve was extracted and the decellularized valve was sutured to the pulmonary valve annulus. **(C)** After complete suturing, the decellularized valve shows good ex vivo dimensional fit in the RVOT orifice.

The decellularized valves appeared slightly larger in diameter due to the decellularization process. On surgical examination, the decellularized pulmonary root performed well, showed no marked fragility, readily supported suture with Surjet 4.0 monofilament thread, and fitted well into the valvular annulus (Figure 8).

## 4 Discussion

This modified decellularization protocol proved effective in removing cellular material from ovine lamb pulmonary valves, as confirmed by histological assessment and DNA quantification, demonstrating an over 90% reduction in nuclear material across all tested valvular regions. These results align with current cellular clearance standards – (DNA reduction greater than 90% and the absence of cellular nuclei on histological examination) (Tzavellas et al., 2025; Breitenstein et al., 2026).

The presence of minimal residual DNA material is expected to trigger only minimal immunogenicity and inflammatory responses, thereby supporting further potential *in vivo* applications (Al Hussein et al., 2025). Because we did not perform direct immunological assays, such as quantification of residual xenogeneic antigens (e.g., α-Gal), assessment of host inflammatory response, or macrophage polarization, we describe the scaffolds as decellularized and DNA-depleted rather than non-immunogenic. Residual immunogenicity will be specifically addressed in future *in vivo* studies.

Compared with previous studies on adult ovine pulmonary valves (Sierad et al., 2015; Kasravi et al., 2023), this protocol demonstrates a short, efficient decellularization process for small, juvenile tissue, delivering rapid, ready-to-use decellularized heart valves.

The hemodynamic assessment in this study demonstrated that decellularized pulmonary valves exhibited physiological valvular function under simulated pulmonary conditions. The absence of statistically significant differences in maximal opening area between the studied group and the fresh valves indicates that the used protocol did not significantly alter the mechanical behavior or structural integrity of the valve. Literature indicates that appropriate decellularization protocols maintain physiological leaflet kinematics and hydrodynamic performance (Waqanivavalagi et al., 2020).

Moreover, qualitative echocardiography showed that the valvular competence remained intact, with complete cusp opening and no evidence of regurgitant jets on color Doppler imaging. These results illustrate that the geometry between the leaflets, Valsalva sinuses and arterial wall remained intact after the cell removal protocol.

The absence of statistically significant differences in maximal velocity, transvalvular pressure gradients and velocity-time integrals indicates an overall preservation of transvalvular flow dynamics. Maintaining a near-physiological state is a critical condition for the new generation of decellularized valves.

The cytocompatibility tests prove short-term cell viability on the decellularized valves. Both fibroblasts and endothelial cells demonstrated normal proliferation and adhesion to all valvular structures over a 14-day incubation time interval, suggesting that cell-attachment components of the extracellular matrix were preserved and that no cytotoxic residues remained after decellularization. However, the Live/Dead assay provides evidence of cytocompatibility only. These data do not demonstrate successful recellularization, endothelialization, or functional tissue regeneration and quantitative evaluation of these processes remain to be demonstrated in future investigations.

The surgical assessment of the decellularized pulmonary roots demonstrated positive handling qualities. Although decellularized valves appeared slightly larger in diameter, this may be related to surgical dissection from the valvular annulus and potential alterations in tissue hydration following the decellularization protocol. During the implant manipulation, the valves showed good mechanical characteristics with no signs of increased frailty. These aspects are important from a translational perspective, as their handling properties are critical for their use in reconstructive cardiac surgery.

The decellularized scaffolds regenerative potential derives primarily from the preserved three-dimensional extracellular matrix, serving as a passive support and secondarily serving as a bioactive, instructive template for host-cells repopulation. Beyond providing mechanical support, the retained matrix presents adhesion ligands and acts as a reservoir of matrix-bound biochemical cues that regulate the adhesion, migration, proliferation, and differentiation of infiltrating cells (Liu et al., 2024). Preservation of both this architecture and its major constituents (collagen, elastin, glycosaminoglycans, laminin, and fibronectin) is therefore considered a key determinant of successful *in situ* remodeling of tissue-engineered heart valves (Fioretta et al., 2021). This current work did not assess these matrix components, and the above reflects the published literature.

Integrin-mediated signaling has been reported to control cellular adhesion, migration, and phenotype, and is believed to influence cell repopulation of the matrix. This interaction may also be modulated by biophysical cues: once implanted in the pulmonary position, the valve is exposed to cyclic hemodynamic loading, and mechanotransduction is thought to translate this mechanical environment into changes in interstitial-cell behavior and in the balance between matrix synthesis and degradation (Poulis et al., 2022). Scaffolds that retain both the structure and the mechanical properties of the native matrix may therefore be more likely to support physiological cell–matrix cross-talk and adaptive remodeling *in vivo* (Fioretta et al., 2021).

Immune recognition of the implant is believed to be an integral part of the remodeling process. Macrophages have been reported to interact with collagen and fibronectin through integrin and non-integrin-mediated pathways, and the phenotype they adopt may determine whether the outcome is constructive remodeling or fibrosis (Poulis et al., 2022). In children, this process would additionally need to keep pace with somatic growth. The scaffold has to remain competent while growing with the recipient, an expectation that current substitutes do not meet, and one that has yet to be demonstrated for decellularized valves *in vivo* (Fioretta et al., 2021). This current work did not assess immunological and remodeling processes, and the above reflects the published literature.

### 4.1 Limitations

A limitation of this study is that ECM validation relied on histology, DNA quantification, and functional (hemodynamic) performance, which demonstrate effective decellularization and preservation of the three-dimensional architecture but do not quantify individual matrix components. As the composition and integrity of the ECM critically determine the biological and mechanical behavior of tissue-engineered heart valves, further studies will include Masson’s and Movat’s pentachrome staining, Alcian Blue quantification of glycosaminoglycans, a hydroxyproline assay for collagen content, and immunohistochemistry for laminin and fibronectin, to confirm quantitative preservation of the extracellular matrix.

Furthermore, the functional assessment in this study was limited to hemodynamic evaluation under simulated physiological pulmonary conditions, which reflects acute valve competence and opening dynamics but not the intrinsic mechanical properties of the tissue. Direct mechanical testing (uniaxial or biaxial tensile testing, elastic modulus determination, and fatigue resistance of leaflets and walls) was not performed and represents an important direction for future work, as these properties are key determinants of long-term durability and clinical performance.

Our biological characterization was limited to qualitative Live/Dead viability assessment and did not include quantitative endpoints. Future studies will incorporate quantitative cell proliferation (metabolic and DNA-based assays, together with Ki-67 labeling), assessment of cell infiltration on histological cross-sections, evaluation of phenotype maintenance, and markers of endothelial functionality (e.g., CD31 and von Willebrand factor), to characterize recellularization and endothelialization more rigorously.

Finally, this is an *in vitro* and *ex vivo* proof-of-concept investigation with a limited sample size for the functional comparison and the mock implantation provided only a qualitative evaluation of surgical handling.

## 5 Conclusions

Following an adapted short protocol in the laboratory, sterile decellularized lamb pulmonary valves were obtained, along with over 90% DNA reduction and preserved three-dimensional extracellular matrix micro-architecture.

When exposed to the pulmonary hemodynamic parameters in the bioreactor, the valves demonstrated physiological behavior without evidence of valvular stenosis or regurgitation. When compared with freshly harvested lamb pulmonary valves, the studied valves showed maximal valvular opening areas, pressure gradients, and velocity–time integrals comparable to those of native valves, and only a minor reduction in mean velocity.

Decellularized valves proved to be a cytocompatible scaffold, maintaining viable cells over 14 days of incubation, exceeding the ISO 10993–5 requirements, supporting the scaffold’s suitability for the recellularization process.

Minor dimensional changes of decellularized valves were noted during surgical implantation, but with no implications in their surgical handling or functional integration in the pulmonary position.

Collectively, these findings validate the decellularized lamb pulmonary valves as stable structures, DNA-depleted and cytocompatible, providing a solid *in vitro* and *ex vivo* proof-of-concept for pediatric regenerative heart valve strategies. Nevertheless, this study does not address long-term durability, *in vivo* remodeling and recellularization, endothelialization, host immune response, or functional integration and growth. These aspects, together with quantitative ECM, biomechanical, and immunological characterization, remain to be demonstrated, and further preclinical validation is required before clinical translation can be considered.

## Data Availability

The original contributions presented in the study are included in the article/supplementary material, further inquiries can be directed to the corresponding author.
